# The value of PMCTA in the diagnosis of coronary atherosclerosis in isolated human hearts

**DOI:** 10.1093/fsr/owad038

**Published:** 2024-01-17

**Authors:** Lei Wan, Jiemin Chen, Zhilu Zhou, Zhengdong Li, Yahui Wang, Donghua Zou, Ningguo Liu, Fengxiang Song, Ping Huang, Zhiyong Zhang, Maowen Wang, Wentao Xia

**Affiliations:** Department of Forensic Pathology, Academy of Forensic Science, Shanghai, China; Department of Radiology, Shanghai Public Health Clinical Center, Fudan University, Shanghai, China; Department of Forensic Pathology, Academy of Forensic Science, Shanghai, China; Department of Radiology, Shanghai Public Health Clinical Center, Fudan University, Shanghai, China; Department of Forensic Pathology, Academy of Forensic Science, Shanghai, China; Department of Forensic Medicine, Guizhou Medical University, Guiyang, China; Department of Forensic Pathology, Academy of Forensic Science, Shanghai, China; Department of Forensic Pathology, Academy of Forensic Science, Shanghai, China; Department of Forensic Pathology, Academy of Forensic Science, Shanghai, China; Department of Forensic Pathology, Academy of Forensic Science, Shanghai, China; Department of Radiology, Shanghai Public Health Clinical Center, Fudan University, Shanghai, China; Department of Forensic Pathology, Academy of Forensic Science, Shanghai, China; Department of Radiology, Shanghai Public Health Clinical Center, Fudan University, Shanghai, China; Department of Forensic Pathology, Academy of Forensic Science, Shanghai, China; Department of Forensic Pathology, Academy of Forensic Science, Shanghai, China

**Keywords:** forensic sciences, forensic pathology, coronary disease, coronary angiography, PMCT, autopsy

## Abstract

Postmortem computed tomography (PMCT) has a limited value in investigating coronary artery disease, despite several obvious advantages over the conventional autopsy. To address this issue, postmortem computed tomography angiography (PMCTA) has been introduced into various studies, where it has been used to investigate natural and unnatural deaths involving vascular damage, occlusion, or other pathologies of the vascular system. To investigate the application value of PMCTA in the diagnosis of coronary artery stenosis in *ex situ* hearts, the water-based contrast media were injected into isolated hearts, scaned, and finally compared with gold standards (autopsy and histology findings of the coronary artery). This study involved 16 subjects from the Academy of Forensic Science who were suspected to have died of sudden death without traumatic injuries. Unenhanced PMCT was performed first, followed by PMCTA using a water-based contrast agent, injected into the coronary arteries of isolated hearts using a self-designed angiography device. The image data were reconstructed into three-dimensional (3D) angiography images using software in the angiography facility. The 3D images were recorded and evaluated by two radiologists and then statistically analysed. The results of PMCTA were consistent with the gold standards for the diagnosis of coronary artery stenosis (*P* > 0.05). However, water-based contrast media can only be used to examine the pathological changes of blood vessels, which may have limitations in the diagnosis of causes of death such as myocardial oedema. PMCTA can be used as a new method to evaluate the degree of coronary atherosclerosis in addition to traditional autopsy. The 3D reconstruction technique reveals the coronary artery lesions more objectively and vividly and provides the opportunity to re-read the data at any time.

**Key points:**

- The methods and parameters for coronary angiography in isolated human hearts were standardized based on the previous researcher.
- PMCTA in isolated human hearts is including the 3D reconstruction technique that reveals the coronary artery lesions more objectively and vividly, and provides the opportunity to re-read the data at anytime.
- PMCTA could only be used to examine the pathological changes of blood vessels, which might have limitations for the diagnosis of causes of death.
- PMCTA in isolated human hearts can be viewed as an auxiliary method for establishing the cause of death, which can provide an assessment of degree and extent of arterial stenosis and accurately help determine the abnormal location.

## Introduction

According to statistics, cardiovascular diseases are the most common cause of death in humans [1]. Coronary artery disease (CAD) is the most common type of disease that causes sudden cardiac death (SCD). In traditional autopsy investigations, a complete autopsy including histological and immunohistochemical staining is the gold standard for determining the cause of death in such cases [2]. On the other hand, postmortem computed tomography (PMCT) has been increasingly used in forensic pathology as a tool to assess the cause of death. Currently, some forensic centres routinely perform PMCT scans before the autopsy [3, 4].

In practice, because the density of the vascular system is similar to that of muscle, it is difficult to investigate the vessels with multidetector computed tomography (MDCT). This is the reason why MDCT has disadvantages of limited soft tissue contrast and low imaging ability of the vascular system [5]. To address this deficiency [6–8], postmortem computed tomography angiography (PMCTA) uses different contrast agents and devices to improve the diagnostic value, such as detecting coronary lesions and bleeding sources. At the same time, PMCTA is considered to be an effective method for the diagnosis of coronary artery stenosis, iatrogenic cerebral infarction, cerebral arteriovenous malformations, and other vascular diseases [9–11].

Inokuchi et al. [12] performed PMCTA on 38 isolated hearts, and the results showed that PMCTA in isolated hearts was helpful to detect artery disease, guide subsequent coronary artery dissection, and objectively and accurately estimate the scope and degree of stenosis. However, there are some drawbacks in PMCTA imaging, such as the possibility of misinterpreting postmortem blood clots as intravascular thrombosis [13]. Also, if the perfusion parameters are not adequate, artefacts may appear [14]. Many forensic pathologists may have paid less attention to isolated single-organ angiography at autopsy, but PMCTA will still be useful if we view it as one of the auxiliary tools for autopsy [12]. Notably, PMCTA performed in a single organ can remove postmortem blood clots by slow flushing and filling with a sufficient volume of contrast agent to make the entire vascularized system visible and reduce artifacts [15]. In view of the above condition, this study compared the results of isolated cardiac PMCTA with autopsy and histology results to analyse the application value of PMCTA in the diagnosis of coronary arteriosclerotic heart disease (AHD) stenosis.

## Materials and methods

### Subjects

A total of 16 adult corpses (12 males, 4 females) without obvious fatal trauma were selected from the Academy of Forensic Science (AFS) from 2016 to 2020, aged between 20 and 68 years (the average age was around 43.4 years). The inclusion criteria were the following: suspected sudden cardiac death and no macroscopic extra-cardiac cause of death was found. Autopsy, histology, and radiological procedures did follow the standards and protocols applied in our Institute. The relatives’ written permission was obtained for the conduct of PMCT, PMCTA, and autopsy on 16 cadavers (in China, the relatives’ consent is required for autopsies except in criminal cases).

### Multidetector computed tomography scanning and image processing parameters

Unenhanced PMCT scans were performed within 1 h of the cadaver’s arrival at the Institute. The cadavers and isolated hearts were scanned with a 40-slice MDCT system (SOMATOM Definition AS; Siemens Medical Solutions, Munich, Germany). The following settings were used for raw data acquisition: voltage, 120 kV; current 240 mA; collimation, 6.0 × 1.0 mm^2^. The RadiAnt DICOM Viewer software (Poznań, Poland) was used for imaging evaluation, and the images were reconstructed at the slice thicknesses of 5.0 and 0.625 mm, respectively, with the thickness of each slice increased by half. The image review and three-dimensional (3D) reconstructions were performed on a CT workstation (Syngo Imaging XS; Siemens Medical Solutions, Forchheim, Germany).

### Perfusion of contrast agent

**Figure 1:**
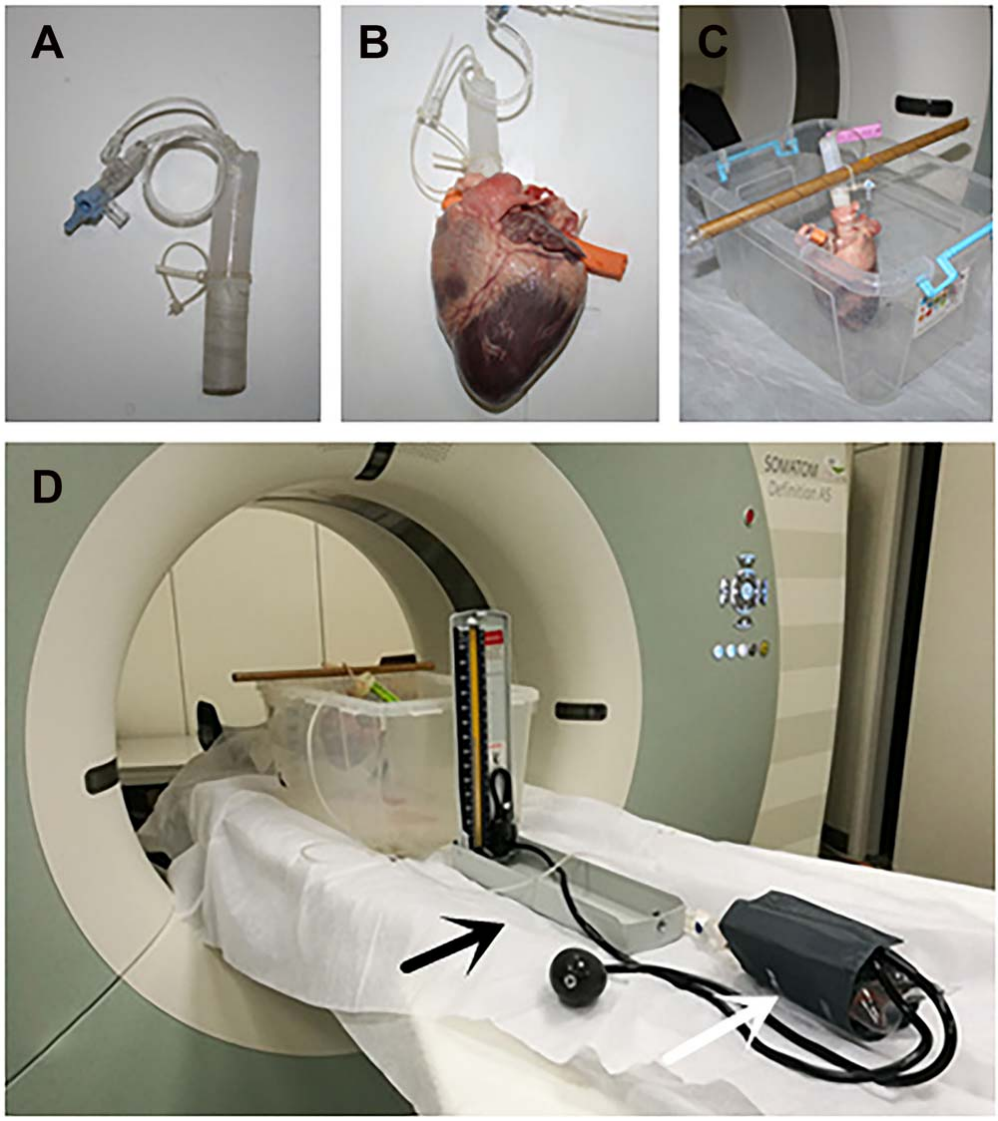
The angiography device. (A) Self-modified design of the three-way valve. (B) Self-modified design of the three-way valve connected to the aorta. (C) The heart was placed on the fixing device. (D) Self-modified design of three-way valve was connected to the sphygmomanometer. The black arrow indicates the sphygmomanometer; the white arrow indicates that a plastic bag of contrast agent is wrapped in the cuff.

After unenhanced PMCT, the heart was completely removed at autopsy, and the aorta was excised 5–8 cm above the heart. Based on previous studies on isolated porcine heart coronary angiography [11], a self-designed and improved isolated cardiac coronary angiography device was adopted. The improved imaging device consists of a sphygmomanometer (a new unit added to the previous device), a self-modified three-way valve and catheter, and a plastic saline bag (Figure 1). Meglumine diatrizoate was commonly used in clinical practice as a contrast agent, and it was relatively easy to obtain. Although water-soluble contrast agents tend to extravasate from the vascular wall, in this study, the time from injection to image acquisition was too short to create significant artefacts due to this phenomenon. First, postmortem blood clots and air were drained out by using a syringe to slowly inject water into the heart. As stated by Inokuchi et al. [12], this method of perfusion would not damage an existing thrombus unless the injection pressure severely exceeds vital pressure. The following steps were carried out: (a) The three-way valve was inserted into the aorta ~2 cm above the coronary sinus, and then the valve and the aorta were fixed tightly. In addition, the aorta and pulmonary vein were ligated. (b) The heart was placed on a fixing device (a plastic bucket or plastic box), and a syringe was used to aspirate the air in the heart cavity from the other channel of the connecting device (channel 1), and then channel 1 was clamped. (c) Channel 2 was then opened and connected to a plastic bag, wrapped in the cuff of the sphygmomanometer, filled with a 10:1 ratio of water-soluble septoglycerin and saline (0.9%) contrast agent. After perfusion, ~80–100 mL of contrast media were injected to maintain the sphygmomanometer at 16.0–20.0 kPa (120–150 mmHg). The CT scan was performed immediately after the sphygmomanometer reading stabilized, and 3D reconstruction was completed after the scan.

The traditional autopsy was performed after the PMCTA examination. During the autopsy, internal and external examinations of the body and heart were performed. The three branches of the coronary arteries were inspected along their long axis at an interval of 2 mm, and tissue sections were made. The diagnosis results of PMCTA were compared with the results of the autopsy, focusing on the degree of coronary arteries stenosis.

### Evaluation of methods and criteria

The effect of contrast imaging was evaluated by two radiologists—a forensic radiologist with 20 years of experience and a clinical radiologist with 30 years of experience. The degree of coronary artery stenosis (percentage reduction in the lumen diameter) was quantitatively evaluated by referring to the normal vessel diameter at the proximal segment of the coronary artery stenosis. According to the vascular filling and lumen diameter shown by angiography (recording the narrowest part of the coronary artery), the evaluation method and standard of the degree of coronary artery stenosis in the isolated heart were judged, and the degree of coronary artery filling and diameter were diagnosed according to the 2D and 3D reconstructed images obtained by PMCTA. The degree of coronary artery stenosis was divided into four grades: severe (≥75%), moderate (50%–74%), mild (25%–49%), and no obvious stenosis (<25%). The coronary arteries observed included the left anterior descending (LAD), the left main coronary artery, the left circumflex (LCX), and the right coronary artery (RCA).

### Statistical methods

The two-sample Kolmogorov–Smirnov test was used to compare the data of random blocks design with Stata 15.0 software (College Station, TX, USA), and the test level was set at 0.05.

## Results

**Figure 2:**
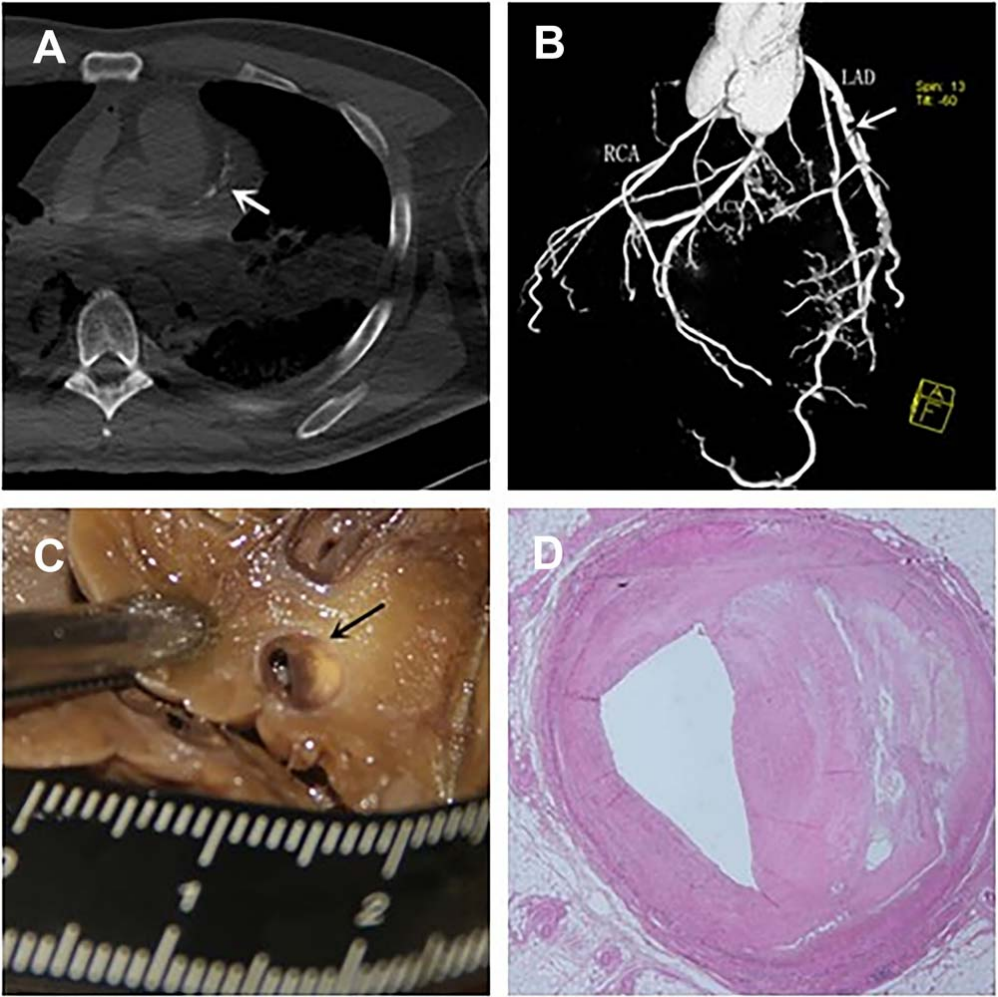
Comparison of angiographic and histopathological images. (A) Multi-slice computer tomography (MSCT) scan indicated calcification of the left main trunk and the left anterior descending branch (white arrow). 3D angiographic images (B) specimens (C) and histopathological images (D) showed the anterior descending branch severe stenosis.

The results of PMCTA and autopsy (including histopathological examination) are shown in Table 1. The degree of stenosis estimated by autopsy was confirmed by histology. PMCTA was consistent with autopsy findings in the diagnosis of coronary atherosclerotic vascular stenosis (*P* = 0.779). PMCTA could display coronary arteries with varying degrees of stenosis, for example, in Case 1 (Figure 2). Positive findings for coronary stenosis were detected in 10 of the 16 cases by PMCTA. The cause of death in five of these 10 cases was diagnosed as related to coronary artery disease (CAD); in the remaining cases, the cause of death was pneumonia, acute haemorrhagic necrotizing pancreatitis, hypertrophic cardiomyopathy leading to heart failure, and intracranial bleeding, respectively. In Case 1 to Case 10, both methods identified mild or higher coronary stenosis. Judgement deviation was found in some cases, for example, RCA of Case 2, LAD of Case 3, and RCA of Case 10. The degree of coronary stenosis by PMCTA was higher than autopsy in Case 2 and Case 3; however, in Case 10, the results were the opposite. Mild stenosis in LAD of Case 13 was detected by autopsy. The rest of the cases showed negative findings of coronary stenosis by both PMCTA and autopsy. Although there are subtle deviations between the two methods in some cases, the overall results were largely consistent.

**Table 1:** The results of postmortem computed tomography angiography (PMCTA) compared with autopsy

Case No.	LAD		LCX		RCA		Cause of death
	Angiography	Autopsy	Angiography	Autopsy	Angiography	Autopsy	
1	+++	+++	++	++	++	++	CAD
2	+++	+++	++	++	+++	++	CAD
3	+++	++	+	+	++	++	CAD
4	+++	+++	++	++	++	++	CAD
5	+++	+++	+	+	+++	+++	CAD
6	+++	+++	++	++	++	++	Pneumonia
7	++	++	+	+	++	++	Acute haemorrhagic necrotizing pancreatitis
8	+	+	++	++	+	+	Rupture of thoracic aorta dissection
9	++	++	+	+	+	+	Hypertrophic cardiomyopathy leading to heart failure
10	+	+	+	+	+	++	Haematencephalon
11	−	−	−	−	−	−	Intoxication
12	−	−	−	−	−	−	Ruptured cerebral aneurysm
13	−	+	−	−	−	−	Hypertrophic cardiomyopathy with myocarditis
14	−	−	−	−	−	−	Intoxication
15	−	−	−	−	−	−	Allergy
16	−	−	−	−	−	−	Electric shock

Note: according to the degree of stenosis: severe (+++), moderate (++), mild (+), and no obvious stenosis (−). LAD: left anterior descending; LCX: the left main coronary artery, the left circumflex; RCA: and the right coronary artery.

## Discussion

As an important aspect of the virtual autopsy, PMCTA technology has been widely reported as a tool for investigating the cadaveric vascular system, especially the coronary arteries [16–18]. Coronary PMCTA studies are mainly divided into two categories: whole-body angiography and single-organ angiography. Inokuchi et al. [12] proposed that *ex situ* cardiac angiography was useful in autopsy if the PMCTA is one of the auxiliary tools. In 2016, Qian et al. [19] performed cerebral angiography on isolated human brains at autopsy and found that single-point angiography showed a cluster of irregular and twisted blood vessels at the bleeding site in the right frontal lobe of the brain. However, with the development of whole-body angiography technology, isolated organ angiography technology is no longer a research hotspot. The reasons for choosing isolated hearts in this study are as follows: First, in routine autopsy, blood vessel slices are usually examined with the naked eye to determine whether there is any abnormality. Abnormal tissues can be sampled for immunohistochemistry, but the heart and brain are both rich in blood vessels, and some abnormal areas may be overlooked for subjective reasons, reducing the chance of a positive result. Second, the PMCT of the cadaver can interfere with the observation of the injury site in cases of haemorrhage. Although PMCTA has been further developed, some scholars believe that targeted coronary angiography technology could be affected by postmortem blood clots, which could hinder the perfusion of coronary arteries, resulting in insufficient coronary artery filling and misdiagnosis as coronary artery stenosis. Although PMCTA of isolated hearts is an invasive examination, it can remove postmortem blood clots and preserve the images. Finally, China has a large and wide population, living in vast areas, rendering the access to imaging devices such as MDCT sometimes difficult. Therefore, postmortem whole-body angiography may not be feasible. PMCTA can improve the accuracy of anatomical determination after obtaining isolated organs. When PMCT or PMCTA is required to assist in the diagnosis of the cause of death, *ex situ* organ transport is more convenient than intact cadaver transportation.

Previous studies established the angiography method and related parameters of isolated porcine heart coronary angiography, which laid the foundation for this study of isolated human heart angiography [12]. In this study, the imaging device was improved based on the previous imaging method of isolated porcine hearts, and the pressure measuring device was added. Improved *ex vivo* cardiac imaging can solve the problem of contrast agent insufficiency. In addition, continuous perfusion can ensure that the blood vessel is permanently filled, preventing the effect of a water-soluble agent to disappear into the surrounding tissues, flatten the lumen of the blood vessels, and affect the judgement of the degree of stenosis.

This study found that PMCTA can clearly show the degree and extent of coronary artery stenosis, and the degree of coronary artery stenosis is highly correlated with histopathological examination. In this study, only the coronary artery RCA in Case 2, the coronary artery LAD in Case 3, and the coronary artery RCA in Case 10 showed differences from histopathological examination, but the differences were small. In Case 2 and Case 3, coronary stenosis was severe on PMCTA results and moderate on histopathological examination. In Case 10, coronary stenosis was mild on PMCTA and moderate on histopathological examination. We hypothesize that it was mainly caused by a smaller heart in this case compared to others, which could have produced a higher perfusion pressure as the injected quantity of contrast agent was the same for all cases. In the future, different perfusion pressure levels could be used for different heart sizes. As in Case 6, PMCTA revealed severe coronary stenosis, which was diagnosed as coronary atherosclerotic heart disease by autopsy. In Cases 7–10, a certain degree of stenosis was found in the coronary arteries. In these cases, misdiagnosis is easy if only coronary PMCTA is performed without an autopsy. In addition, in Cases 11–16, although PMCTA made a correct diagnosis of the degree of coronary artery stenosis, the cause of death also could not be determined without an autopsy. Therefore, the degree of coronary artery stenosis alone is not sufficient to determine the cause of death.

Single-organ PMCTA can accurately locate and determine the stenosis, which is helpful to guide the detection of lesions at autopsy, thus reducing false-negative results [5, 12]. PMCTA allows to observe coronary arteries at multiple angles and in different ways at the same time. Moderne software (Miami, FL, USA) can even calculate the degree of coronary artery stenosis. In the autopsy, the coronary arteries are crossed every 2 mm along their long axis. Compared with PMCTA, visual incision inspection and empirical judgement of the degree of vascular stenosis are easy to miss or misdiagnose. Moreover, in our study, removing the heart from the body was destructive, but the PMCTA technology converts information into images and saves it through CT scans, which can be reused, transmitted remotely to expert consultation, and provide a relatively objective diagnosis. The lesion of the coronary artery can also be presented in 3D form as intuitive evidence in court [7]. Further research on the application of PMCTA in coronary atherosclerotic vascular stenosis is needed to promote its application in practical forensic pathology examination. PMCTA technology can achieve objective results, and 3D reconstruction technology can show the position, shape, range, and degree of stenosis of the left and right main coronary arteries and other branches. PMCTA can improve the comprehensive evaluation ability of vascular diseases in the deceased; accurately determine the degree of coronary artery stenosis; and diagnose coronary artery stenosis, vascular dysplasia, aneurysms, and other lesions. PMCTA provides a reference for the localization of small lesions and provides a basis for the final inspection and identification of the lesion during autopsy. As a new technology, PMCTA has shown great potential in the field of forensic pathology. Although PMCTA cannot completely replace autopsy, it can be used as an adjunct to traditional autopsy.

In conclusion, this study used a self-developed and effective method for isolated human heart PMCTA angiography. Performing PMCTA on an isolated heart is useful for the auxiliary diagnosis of coronary artery stenosis in cadavers. The drawback is that we used the same pressure for contrast injection in this work, and problems with insufficient or excessive contrast can occur in large or small hearts. We believe that in the future, the injection pressure should be adjusted according to the sizes of the investigated organ.

## Authors’ contributions

Yahui Wang and Jiemin Chen was responsible for the methodology. Maowen Wang operated the software. Zhengdong Li validated the results and provided the resources. Donghua Zou was responsible for the formal analysis. Fengxiang Song contributed to the case investigation. Ningguo Liu was responsible for the data curation. Lei Wan wrote the original draft. Zhilu Zhou and Lei Wan wrote the review and edited the manuscript. Ping Huang contributed to the visualization. Zhiyong Zhang was responsible for the supervision. Wentao Xia contributed to the project administration. Zhiyong Zhang and Wentao Xia managed the funding. All authors have read and agreed to the published version of the manuscript.

## Compliance with ethical standards

The relevant ethical approval was obtained from the Ethics Committee of the Academy of Forensic Science, China. The relatives’ written permission was obtained for the conduct of PMCT, PMCTA, and autopsy on 16 cadavers (in China, the relatives’ consent is required for autopsies except in criminal cases).

## Disclosure statement

None declared.

## Funding

This study was supported by the National Key Research and Development Plan [grant number 2016YFC0800702], Council of National Science Foundation of China [grant numbers 81401559 and 81571851], Scientific and Technological Key Project of Shanghai Municipality [grant number 17DZ2273200], Central Research Institute Public Project [grant numbers GY2021Z-1], and the Basic Scientific Research Services of the Academy of Forensic Science, Ministry of Justice, PR China [grant numbers GY2018G-2, GY2021G-8, and GY2016G-4].
